# Investigation of a novel therapeutic approach in multiple sclerosis

**DOI:** 10.1186/1753-6561-7-S1-O6

**Published:** 2013-01-30

**Authors:** Biró Eniko, A Folyovich, B Vásárhelyi, G Toldi

**Affiliations:** 1 First Department of Pediatrics Budapest, Hungary; 2 Szent János Hospital Budapest, Hungary

## Background

Multiple sclerosis (MS) is an autoimmune disease affecting the central nervous system that generally develops in young adults. T lymphocytes play a key role in the pathogenesis of MS. The main types of lymphocytes are CD4 (Thelper) and CD8 (Tkiller) cells. CD4 cells can further be divided into Th1 and Th2 cells. Th1 cells are responsible for sustaining the autoimmune response. In contrast, Th2 cells reduce inflammatory reactions. Previous investigations suggested that the inhibition of the Kv1.3 and IKCa1 channels enable the specific modulation of different lymphocyte subsets. The ratio of the above channels is dissimilar in different types of lymphocytes. The inhibition of these channels leads to reduced activation of lymphocytes, and, therefore, to a decrease of the autoimmune reaction. In our investigations we aimed to observe the differences in lymphocyte activation upon the inhibition of the above channels in the Th1, Th2, CD4 and CD8 subsets in MS compared to healthy individuals. Furthermore, we aimed to assess whether inhibition of lymphocyte potassium channels may be a possible target for the future therapy of MS.

## Methods

We used flow cytometry for our investigations. We analyzed samples of 10 healthy individuals and 10 newly diagnosed MS patients not receiving immunomodulatory therapy. First, we separated lymphocytes and stained the Th1, Th2, CD4 and CD8 subsets with cell surface markers. Then, cells were loaded with calcium-sensitive fluorescent dyes and were treated with the blockers of the above-mentioned channels. We activated cells with phytohemagglutinine immediately before the flow cytometry recording. Finally, data were evaluated by a software developed in our laboratory.

## Results

In MS, the reactivity of lymphocytes is increased compared to healthy controls. In MS there was no difference revealed between the investigated subsets upon blocking the IKCa1 channels. The inhibition of Kv1.3 channels in MS decreased the activation of CD8 cells to a higher extent than that of CD4 cells, but such a difference was not detectable between Th1 and Th2 cells.

## Conclusions

Our findings suggest that the activation of lymphocytes can be decreased in MS by blocking the investigated channels. Although limited selectivity can be reached in MS. Accordingly, further investigation is needed to assess how the inhibition of lymphocyte potassium channels modulates the whole immune response in MS.

